# Visceral adiposity index is positively associated with fasting plasma glucose: a cross-sectional study from National Health and Nutrition Examination Survey 2017–2020

**DOI:** 10.1186/s12889-023-15231-8

**Published:** 2023-02-11

**Authors:** Yuhan Qin, Yong Qiao, Dong Wang, Mingkang Li, Zhanneng Yang, Linqing Li, Gaoliang Yan, Chengchun Tang

**Affiliations:** Department of Cardiology, Zhongda Hospital, School of Medicine, Southeast University, Nanjing, 210009 Jiangsu China

**Keywords:** NHANES, Visceral adiposity index, Fasting plasma glucose, Diabetes, Obesity

## Abstract

**Background:**

Visceral adiposity index (VAI) has been recognized as a reliable indicator for visceral adiposity. However, it remains largely unexplored on its association with fasting plasma glucose (FPG). The current study aims to explore the association between VAI and FPG using a representative dataset.

**Methods:**

A cross-sectional study was carried out based on the dataset from National Health and Nutrition Examination Survey (NHANES) 2017–2020. Univariate and Multiple linear regression analysis were performed to explore the relationship between VAI and FPG. Generalized additive model (GAM) and smooth curve fitting analysis were performed to explore the nonlinear relationship between VAI and FPG. Receiver operating characteristic (ROC) analysis was used to evaluate the predictive value of VAI for FPG elevation.

**Results:**

A total of 4437 participants with complete data were finally included in the research. Individuals were divided into 4 quartiles according to the calculated VAI value: Q1 (VAI<0.69), Q2 (0.69 ≤ VAI < 1.18), Q3 (1.18 ≤ VAI < 2.02) and Q4 (VAI ≥ 2.02). FPG significantly increased with the increasing VAI quartile. Multiple linear regression analysis showed VAI was independently positively associated with FPG after adjusting confounding factors. As a continuous variable, an increase of one unit in VAI was correlated with 0.52 mmol/L (95% CI: 0.41–0.63, *p* < 0.0001) higher FPG level. As a categorical variable, 4th VAI quartile group was related to 0.71 mmol/L (95% CI: 0.47–0.95, *p* < 0.001) higher FPG level compared with 1st VAI group. GAM and smooth curve fitting analysis identified the non-linear relationship between VAI and FPG, and 4.02 was identified as the inflection point using two-piecewise linear regression. The positive association between VAI and FPG existed when VAI was lower (β = 0.73, *p* < 0.0001) and higher than 4.02 (β = 0.23, *p* = 0.0063). ROC analysis indicated VAI has a good predictive value for FPG elevation (AUC = 0.7169, 95% CI: 0.6948–0.7389), and the best threshold of VAI was 1.4315.

**Conclusion:**

VAI was an independently risk indicator for FPG, and VAI was nonlinearly positively associated with FPG. VAI had a good predictive value for elevated FPG. VAI might become a useful indicator for risk assessment and treatment of hyperglycemia in clinical practice.

## Background

Data have shown steady increase in diabetes in many countries. The prevalence rate of diabetes in China sharply increased from less than 1% in the 1980s to nearly 11% in 2013 [1]. The estimated overall prevalence of diabetes and prediabetes reached to 12.4 and 38.1% in 2018 according to the nationally cross-sectional survey conducted in mainland China [2]. It is estimated that over 783 million population are expected to develop diabetes by 2045, which is related to as high as 1054 billion USD health expenditures [3]. Impaired fasting glucose (IFG) has been identified as an acknowledged risk factor for Type 2 diabetes mellitus (T2DM) [4]. Moreover, higher fasting plasma glucose (FPG) was closely associated with increased mortality [5], and research finding showed that elevated FPG levels within the normal range were also related to higher risk for T2DM [6].

Among the multi-factors in the pathophysiology of T2DM, obesity has been recognized as an essential contributor. Obesity has become a global health and economic concern due to its increasing prevalence and heavy disease burden [7]. The role of obesity in T2DM is attracting considerable research interest worldwide [8]. Research showed obese subjects with body mass index (BMI)>30 had 7.19 times higher risk for developing T2DM compared with those with normal weight (BMI < 25) [9]. In addition, obesity significantly increased the risk of abnormal FPG (OR = 1.44), and the OR reached 1.84 in individuals with moderate/severe obesity [10]. It is worth noting that excessive visceral fat deposition and ectopic fat were recognized as emerging risk factors for diabetes compared with peripheral deposition of fat [11]. Growing evidence has proved that visceral obesity is associated with worsening of insulin sensitivity [12] and increased risk of getting diabetes [13]. Larger amount of visceral adipose tissue quantified by multiparametric magnetic resonance imaging was correlated with higher risk for developing T2DM [14].

Magnetic resonance-based assessment could accurately assess lean and adipose tissue according to the distinct magnetic properties of fat and water. However, it is not suitable for widespread clinical use because it is expensive and time consuming [15]. Therefore, it is urgent to establish a convenient and cost-effective method for the assessment of visceral obesity. The most commonly used parameter, BMI, can no longer help clinicians evaluate and manage obesity-related health risk solely because this general adiposity indicator is difficult to distinguish between subcutaneous and visceral obesity [16]. In a prospective study included 10,419 Chinese adults, waist circumference (WC) was found strongly correlated with the higher risk for T2DM in comparison with BMI [17]. Act as a simply applicable anthropometric index for abdominal adiposity assessment, WC is recommended in clinical practice to optimize obesity risk stratification [18]. Marco C et al. [19] extrapolated a novel gender-specific indicator termed visceral adiposity index (VAI) for visceral adiposity evaluation based on simple biochemical metabolic and anthropometric indicators, including WC, BMI, triglyceride (TG), and high-density lipoprotein (HDL). VAI is considered as a surrogate indicator of visceral adipose distribution and visceral fat dysfunction, and identified as an independent risk factor for cardiovascular events, cerebrovascular events with higher sensitivity and specificity compared with classic parameters [19–21].

Numerous researches have confirmed the relationship between VAI, diabetes, and diabetic complications. Recently, Zhang reported VAI was an independent risk factor for developing newly diagnosed T2DM and VAI had a strong predictive value for T2DM during 4-year follow-up of 4078 Chinese adults [22]. Another study including 1091 non-diabetic participants showed that VAI was associated with 11% higher risk for developing T2DM after 5-year follow-up [23]. A cohort study enrolling 8948 T2DM patients indicated VAI was dramatically associated with an increased risk for diabetic nephropathy (HR = 1.127; 95% CI: 1.050–1.210) [24]. However, it remains unknown the association between VAI and FPG. Therefore, the present study aims to investigate the relationship between VAI and FPG based on the NHANES database with large sample size, which might contribute to early diagnosis, risk assessment and therapeutic intervention of elevated FPG.

## Methods

### Data source and study population

NHANES is a national representative cross-sectional survey on non-institutionalized population in the US with publicly accessible data, conducted by the National Centers for Disease Control and Prevention, which included demographic, dietary, examination, laboratory, questionnaire and limited data. Datasets from NHANES 2017 to 2020 required for the analysis were obtained from the NHANES website (https://www.cdc.gov/nchs/nhanes/). The written informed consents were obtained from all participants, and the program was approved by the Ethics Review Board of National Center for Health Statistics.

**Fig. 1:**
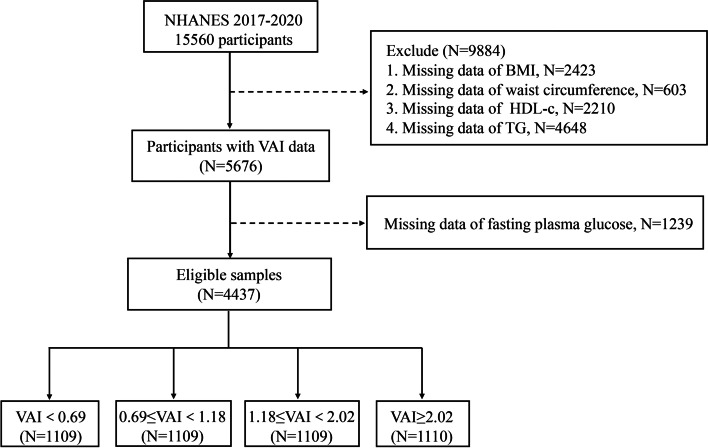
Flow diagram displaying the enrollment of study population

Of 15,560 candidates extracted from the NHANES database, 9884 participants aged≥18 without indispensable information for VAI calculation were excluded (missing data of BMI, *N* = 2423; missing data of waist circumference, *N* = 603; missing data of HDL-c, *N* = 2210 and missing data of TG, *N* = 4648). Individuals without information on fasting plasma glucose (*N* = 1239) were also excluded. Eventually, a total of 4437 participants were enrolled in this study, and the final selected subjects were divided into 4 groups according to the VAI value. The flow diagram of selection process was provided in Fig. 1.

### Anthropometric indexes measurement

High-quality anthropometric measurement data was collected by well-trained NHANES staff following standardized examination protocols, using standardized examination procedures and calibrated equipment. A stadiometer with an adjustable head piece and a fixed vertical backboard was used to measure standing height. Participants were weighed in kilograms using a digital weight scale. The formula weight (kilograms)/square of height (meters) was used to determine BMI values. WC was measured to the closest 1 mm at the end of the normal expiration. Draw a horizontal line just above the right ilium’s uppermost lateral border, and wrap the measuring tape around the waist. Blood pressure (BP) in sitting position was measured in the right arm sing Omron HEM-907XL BP monitor. After a rest of 5 min and triplicate BP determinations with at least 1 min interval were taken. The means of the three BP measurements were used in the following analysis.

### Study variables

The demographics questionnaires were asked by accomplished interviewers using Computer-Assisted Personal Interview (CAPI) system. Participants venous blood samples were collected and examined following the NHANES laboratory protocol. All methods of standard biochemistry were measured on the Roche Cobas 6000 (c501 module) analyzer. More detailed information about analyte methodologies, principles, and operating procedures could be found in the Laboratory Method Files at https://wwwn.cdc.gov/Nchs/Nhanes/. The information on treatment of diabetes, hypertension and hyperlipidemia was provided at the questionnaire data.

The collected anthropometric data (BMI and WC) and biochemical data (TG and HDL) were used for VAI calculation by previous reported formula [19], and the units of WC and BMI were cm and Kg/m^2^, respectively. Both TG and HDL were expressed in mmol/L.

$${\displaystyle \begin{array}{c} Male: VAI=\frac{WC}{39.68+1.88\times BMI}\times \frac{TG}{1.03}\times \frac{1.31}{HDL}\\ {} Female: VAI=\frac{WC}{36.58+1.89\times BMI}\times \frac{TG}{0.81}\times \frac{1.52}{HDL}\end{array}}$$

FPG was measured using the Roche/Hitachi Cobas C311 UV assay. Diabetes was defined as a self-reported history of diabetes, HbA1c level ≥ 6.5% or FPG level ≥ 7.0 mmol/L, according to the 2019 American Diabetes Association criteria [25].

Smoking was defined as smoking over 100 cigarettes during entire lifetime [26]. Moderate and vigorous activity were defined as ‘activity that causes small, and large increase in breathing or heart rate, respectively [27]. For example. Brisk walking was classified as moderate activity, while carrying heavy loads was categorized as vigorous activity. Stoke was self-reported and physician diagnosis using epidemiological data from NHANES [28]. eGFR was calculated through the Chronic Kidney Disease Epidemiology Collaboration (CKD-EPI) equation. Then, CKD were defined according to the KDIGO guidelines [29]: stage 1, urinary albumin-to-creatinine ratio (ACR) ≥ 3 mg/mmol with eGFR ≥90 ml/min/1.73 m^2^; stage 2, ACR ≥ 3 mg/mmol with 60 ml/min/1.73 m^2^ ≤ eGFR ≤89 ml/min/1.73 m^2^; stage 3, 30 ml/min/1.73 m^2^ ≤ eGFR ≤59 ml/min/1.73 m^2^ (with or without ACR ≥ 3 mg/mmol); stage 4, 15 ml/min/1.73 m^2^ ≤ eGFR ≤29 ml/min/1.73 m^2^; and stage 5, eGFR < 15 ml/min/1.73 m^2^.

Covariates in multivariate models might contribute to muddled correlations between VAI and FPG. The following variables were selected as covariates in the current study: age, gender, SBP, DBP, HR, smoking, physical activity, hypertension, hyperlipidemia, stroke, CKD, race, TC, LDL-c, ALT and ALP based on univariate analysis and previous studies.

### Statistical analysis

All statistical analyses were performed using R software and EmpowerStats. Sample weights were adjusted to present nationally representative estimates in all analyses according to the stratified, multistage probability sampling design [30]. Continuous and categorical variables were presented as mean ± standard error and frequencies (percentages), respectively. Participants were equally divided into 4 quartiles according to the calculated VAI value: Q1 (VAI < 0.69), Q2 (0.69 ≤ VAI < 1.18), Q3 (1.18 ≤ VAI < 2.02) and Q4 (VAI ≥ 2.02). One-way analysis of variance and Kruskal-Wallis tests were used for comparison the differences of characteristics between 4 VAI groups for continuous variables and chi-square tests were utilized for categorical variables. The independent relationship between exposure factors and FPG was calculated using univariate analysis and multiple linear regression model. The association between VAI and FPG was expressed using 3 models with different adjustment for confounding factors: a crude model; minimally adjusted model: adjusted for age and gender; fully adjusted model: adjusted for age, gender, SBP, DBP, HR, smoking, physical activity, hypertension, hyperlipidemia, stroke, CKD, race, TC, LDL-c, ALT and ALP. These confounders were selected on the basis of their associations with FPG or an alteration in effect estimate of over 10% [31]. Non-linear relationships between VAI and fasting plasma glucose were explored using generalized additive model (GAM) and smooth curve fitting. Furthermore, we calculated the inflection points using two-piecewise linear regression model. The receiver operating characteristic (ROC) curve was utilized to evaluate the predictive potential of VAI for FPG elevation. The best threshold was determined according to the sum of sensitivity and specificity. A two-sided *P* < 0.05 was considered statistically significant. Sample weighting was applied for the unequal probability of individual sampling, resulting from complex multistage survey design.

## Results

### Baseline characteristics of the participants

Table 1 described the baseline characteristics of the study population. Four thousand four hundred thirty-seven individuals were divided into 4 groups according to the VAI value. 2175 (49.02%) were males, and the average age of included subjects was 44.41 ± 18.94. Subjects in the 4th VAI quartile group had significantly higher levels of age, anthropometric indexes (including BMI, SBP, DBP, HR, WC and hip circumference), WBC, neutrophil, hemoglobin, platelet, FPG, HbA1c, TG, TC, HLD-c, LDL-c, globulin, ALT, γGT, and uric acid. It is worth noting that FPG increased rapidly from 5.55 to 6.70 mmol/L with the increasing VAI quartiles (*p* < 0.0001). The percentage of smoking, hypertension, hyperlipidemia, diabetes, stroke, and CKD history, and the proportion of taking glucose-lowering, antihypertensive and lipid-lowering drugs also rose with the increasing of VAI quartiles. However, subjects with higher VAI value presented lower levels of albumin and total bilirubin, percentage of male and vigorous physical activity were less frequent. Additionally, different race distribution was found, as the VAI quartile increased, the proportion of Mexican American, Other Hispanic and Non-Hispanic White increased, while Non-Hispanic Black decreased. No statistical significances were observed in other variables among the VAI quartile groups.

**Table 1:** Baseline characteristics of population with different VAI levels

Variables	Q1 (VAI < 0.69) (*n* = 1109)	Q2 (0.69 ≤ VAI < 1.18) (*n* = 1109)	Q3 (1.18 ≤ VAI < 2.02) (*n* = 1109)	Q4 (VAI ≥ 2.02) (*n* = 1110)	*P* value
Age (year)	38.03 ± 18.82	44.30 ± 19.74	45.62 ± 19.14	49.19 ± 16.38	< 0.0001
Male (n, %)	607 (54.73%)	546 (49.23%)	505 (45.54%)	517 (46.58%)	0.0003
BMI (kg/m^2^)	24.95 ± 5.21	27.95 ± 6.71	30.64 ± 7.54	32.34 ± 7.10	< 0.0001
SBP (mmHg)	117.16 ± 16.32	119.48 ± 17.03	120.90 ± 18.36	122.12 ± 16.10	< 0.0001
DBP (mmHg)	70.17 ± 10.99	72.34 ± 10.98	74.03 ± 11.48	76.37 ± 11.25	< 0.0001
HR (bpm)	66.18 ± 11.10	66.96 ± 10.93	68.76 ± 10.84	70.37 ± 11.96	< 0.0001
Waist circumference (cm)	86.74 ± 13.87	95.69 ± 16.02	102.79 ± 17.63	107.76 ± 15.54	< 0.0001
Hip circumference (cm)	99.43 ± 11.05	104.80 ± 13.53	109.41 ± 15.00	112.07 ± 14.54	< 0.0001
Smoking (n, %)	342 (30.84%)	354 (31.92%)	425 (38.32%)	501 (45.14%)	< 0.0001
Drinking (n, %)	111 (10.01%)	123 (11.09%)	137 (12.35%)	136 (12.25%)	0.0982
Physical activity					0.0048
Mild work (n, %)	427 (38.50%)	423 (38.14%)	430 (38.77%)	415 (37.39%)	
Moderate work (n, %)	426 (38.41%)	425 (38.32%)	423 (38.14%)	455 (40.99%)	
Vigorous work (n, %)	256 (23.08%)	261 (23.53%)	256 (23.08%)	240 (21.62%)	
VAI	0.49 ± 0.13	0.93 ± 0.14	1.51 ± 0.23	3.58 ± 2.78	< 0.0001
Hypertension (n, %)	231 (20.83%)	317 (28.58%)	408 (36.79%)	498 (44.86%)	< 0.0001
Hyperlipidemia (n, %)	189 (17.04%)	293 (26.42%)	370 (33.36%)	527 (47.48%)	< 0.0001
Diabetes (n, %)	57 (5.14%)	107 (9.65%)	165 (14.88%)	273 (24.59%)	< 0.0001
Stroke (n, %)	32 (2.89%)	37 (3.34%)	58 (5.23%)	52 (4.68%)	0.0374
CKD (n, %)	25 (2.25%)	31 (2.80%)	43 (3.88%)	52 (4.68%)	0.007
Race (n, %)					< 0.0001
Mexican American	101 (9.11%)	140 (12.62%)	165 (14.88%)	194 (17.48%)	
Other Hispanic	78 (7.03%)	116 (10.46%)	112 (10.10%)	138 (12.43%)	
Non-Hispanic White	325 (29.31%)	346 (31.20%)	376 (33.90%)	445 (40.09%)	
Non-Hispanic Black	408 (36.79%)	315 (28.40%)	260 (23.44%)	127 (11.44%)	
Other Race: Including Multi-Racial	197 (17.76%)	192 (17.31%)	196 (17.67%)	206 (18.56%)	
WBC (10^9^/L)	6.03 ± 1.88	6.49 ± 1.72	6.98 ± 2.10	7.52 ± 2.07	< 0.0001
Neutrophil (10^9^/L)	3.46 ± 1.61	3.74 ± 1.38	4.07 ± 1.56	4.46 ± 1.65	< 0.0001
Hb (g/L)	14.18 ± 1.43	14.29 ± 1.49	14.28 ± 1.41	14.42 ± 1.47	0.0007
PLT (10^9^/L)	232.01 ± 55.48	244.82 ± 63.69	250.53 ± 64.15	250.12 ± 59.15	< 0.0001
FPG (mmol/L)	5.55 ± 0.74	5.73 ± 1.13	5.96 ± 1.33	6.70 ± 2.59	< 0.0001
HbA1c (%)	5.32 ± 0.49	5.49 ± 0.66	5.65 ± 0.78	6.00 ± 1.29	< 0.0001
TG (mmol/L)	0.53 ± 0.15	0.82 ± 0.20	1.15 ± 0.26	2.19 ± 1.40	< 0.0001
TC (mmol/L)	4.41 ± 0.94	4.59 ± 0.96	4.76 ± 1.07	5.08 ± 1.14	< 0.0001
HDL-c (mmol/L)	1.73 ± 0.42	1.47 ± 0.35	1.30 ± 0.28	1.10 ± 0.22	< 0.0001
LDL-c (mmol/L)	2.44 ± 0.75	2.74 ± 0.82	2.93 ± 0.92	3.01 ± 0.98	< 0.0001
Total protein (g/L)	71.24 ± 4.32	70.89 ± 4.16	71.10 ± 4.42	70.84 ± 4.11	0.0897
Albumin (g/L)	41.96 ± 3.26	41.00 ± 3.12	40.34 ± 3.38	40.06 ± 3.31	< 0.0001
Globulin (g/L)	29.28 ± 3.90	29.88 ± 4.07	30.76 ± 4.06	30.78 ± 3.99	< 0.0001
ALT (U/L)	18.29 ± 18.37	21.04 ± 15.35	22.04 ± 17.49	25.69 ± 17.21	< 0.0001
AST (U/L)	21.39 ± 14.37	21.73 ± 12.46	20.96 ± 11.79	21.96 ± 10.54	0.2395
ALP (U/L)	81.64 ± 56.36	82.88 ± 47.34	83.84 ± 41.54	80.95 ± 31.34	0.4266
γGT (U/L)	19.98 ± 22.77	28.09 ± 67.54	27.45 ± 32.27	33.54 ± 32.67	< 0.0001
LDH (U/L)	154.10 ± 36.11	156.46 ± 33.47	156.96 ± 29.95	154.08 ± 36.05	0.0883
Total bilirubin (μmol/L)	9.66 ± 6.72	9.18 ± 5.79	8.26 ± 4.78	7.53 ± 4.07	< 0.0001
Creatinine (μmol/L)	74.10 ± 21.65	75.71 ± 28.50	74.87 ± 29.87	76.81 ± 44.43	0.2234
Uric acid (μmol/L)	291.47 ± 72.49	312.81 ± 76.08	330.40 ± 85.78	341.87 ± 85.82	< 0.0001
Glucose-lowering drugs (n, %)	39 (3.52%)	93 (8.39%)	135 (12.17%)	233 (20.99%)	< 0.0001
Antihypertensive drugs (n, %)	169 (15.24%)	235 (21.19%)	329 (29.67%)	383 (34.50%)	< 0.0001
Lipid-lowering drugs (n, %)	126 (11.36%)	169 (15.24%)	233 (21.01%)	307 (27.66%)	< 0.0001

*Abbreviations*: *BMI* Body mass index, *SBP* Systolic blood pressure, *DBP* Diastolic blood pressure, *HR* Heart rate, *VAI* Visceral adiposity index, *CKD* Chronic kidney disease, *WBC* White blood count, *Hb* Hemoglobin, *PLT* Platelet, *FPG* Fasting plasma glucose, *HbA1c* Hemoglobin A1c, *TG* Triglyceride, *TC* Total cholesterol, *HDL-c* High-density lipoprotein-cholesterol, *LDL-c* Low-density lipoprotein-cholesterol, *ALT* Alanine transaminase, *AST* Aspartate transaminase, *ALP* Alkaline phosphatase, *γGT* Gamma-glutamyl transferase, *LDH* Lactate dehydrogenase

### Univariate analysis for FPG

Univariate linear analysis was performed to evaluate the relationship between the variables and FPG. As shown in Table 2, age, male, SBP, DBP, HR, WC, hip circumference, smoking, hypertension, hyperlipidemia, diabetes, stroke, CKD, TG, TC, HDL-c, LDL-c, ALT and ALP were all positively associated with FPG level (*p* < 0.05). Vigorous physical activity and HDL-c were negatively related to FPG level (*p* < 0.05). Compared with Mexican American, we observed a significant negative correlation between Non-Hispanic White and FPG, whereas no significant association was found between moderate physical activity, AST, total bilirubin, other races and FPG. Of note, VAI was significantly positively correlated with FPG (β = 0.24, 95% CI: 0.21–0.27, *p* < 0.0001).

**Table 2:** Univariate analysis for fasting plasma glucose

Variables	Statistics	β (95%CI)	*P* value
Age	45.21 ± 20.40	0.02 (0.02, 0.03)	< 0.0001
Gender			< 0.0001
Female	2262 (50.98%)	Reference	
Male	2175 (49.02%)	0.32 (0.22, 0.42)	
BMI	29.08 ± 7.46	0.05 (0.04, 0.05)	< 0.0001
SBP	120.00 ± 19.95	0.02 (0.02, 0.02)	< 0.0001
DBP	72.04 ± 12.42	0.02 (0.01, 0.02)	< 0.0001
HR	71.18 ± 12.29	0.02 (0.02, 0.03)	< 0.0001
Waist circumference	98.08 ± 18.17	0.03 (0.02, 0.03)	< 0.0001
Hip circumference	105.91 ± 14.94	0.02 (0.01, 0.02)	< 0.0001
Smoking	1622 (36.56%)	0.28 (0.18, 0.39)	< 0.0001
Physical activity			
Mild work (n, %)	1695 (38.20%)	Reference	
Moderate work (n, %)	1729 (38.97%)	−0.02 (−0.12, 0.08)	0.7043
Vigorous work (n, %)	1013 (22.83%)	−0.12 (− 0.23, − 0.00)	0.0493
VAI	1.65 ± 2.01	0.24 (0.21, 0.27)	< 0.0001
Hypertension	1454 (32.77%)	0.90 (0.80, 1.01)	< 0.0001
Hyperlipidemia	1379 (31.08%)	0.60 (0.49, 0.70)	< 0.0001
Diabetes	602 (13.57%)	3.22 (3.08, 3.36)	< 0.0001
Stroke	179 (4.03%)	0.59 (0.30, 0.89)	< 0.0001
CKD	151 (3.40%)	0.57 (0.26, 0.88)	0.0003
Race			
Mexican American	600 (13.52%)	Reference	
Other Hispanic	444 (10.01%)	−0.17 (−0.42, 0.07)	0.1632
Non-Hispanic White	1492 (33.63%)	−0.23 (− 0.40, − 0.06)	0.0089
Non-Hispanic Black	1110 (25.02%)	−0.18 (− 0.39, 0.04)	0.1134
Other Race: Including Multi-Racial	791 (17.83%)	−0.11 (− 0.34, 0.11)	0.3144
TG	1.17 ± 1.02	0.44 (0.39, 0.49)	< 0.0001
TC	4.65 ± 1.06	0.09 (0.04–0.15)	0.0009
HDL-c	1.38 ± 0.40	− 0.82 (− 0.94, − 0.69)	< 0.0001
LDL-c	2.73 ± 0.91	0.05 (0.01, 0.09)	0.012
ALT	21.34 ± 19.27	0.01 (0.01, 0.01)	< 0.0001
AST	21.56 ± 14.80	0.01 (−0.00, 0.01)	0.2828
ALP	88.07 ± 49.74	0.00 (0.00, 0.00)	< 0.0001
Total bilirubin	8.38 ± 5.10	0.00 (−0.01, 0.01)	0.3773

*Abbreviations*: *BMI* Body mass index, *SBP* Systolic blood pressure, *DBP* Diastolic blood pressure, *HR* Heart rate, *VAI* Visceral adiposity index, *CKD* Chronic kidney disease, *TG* Triglyceride, *TC* Total cholesterol, *HDL-c* High-density lipoprotein-cholesterol, *LDL-c* Low-density lipoprotein-cholesterol, *ALT* Alanine transaminase, *AST* Aspartate transaminase, *ALP* Alkaline phosphatase

### Association between VAI and FPG in different models

Multiple linear regression model was used to evaluate the independent relationship between VAI and FPG. Table 3 displayed the β (95% CI) of VAI for FPG in different models. Higher VAI levels were remarkably associated with increased FPG levels. As a continuous variable, an increase of one unit in VAI was associated with 0.24 (95% CI: 0.21–0.27, *p* < 0.0001), 0.21 (95% CI: 0.19–0.24, *p* < 0.0001), and 0.52 mmol/L (95% CI: 0.41–0.63, *p* < 0.0001) higher FPG level, respectively, in crude model, minimally adjusted and fully adjusted model. As a categorical variable, 4th VAI quartile group was associated with 0.71 mmol/L (95% CI: 0.47–0.95, *p* < 0.001) higher FPG level after controlling all the potential confounding factors in fully adjusted model compared with participants with lowest FPG level in Q1.The trend test remained significant (*p* < 0.001).

**Table 3:** Relationship between VAI and fasting plasma glucose in different models

Exposure	Crude Model		Model I		Model II	
	β (95%CI)	*P* value	β (95%CI)	*P* value	β (95%CI)	*P* value
VAI	0.24 (0.21, 0.27)	< 0.0001	0.21 (0.19, 0.24)	< 0.0001	0.52 (0.41, 0.63)	< 0.0001
VAI (Quartile)						
Q1	Reference		Reference		Reference	
Q2	0.18 (0.04, 0.32)	0.0109	0.08 (−0.06, 0.21)	0.2612	0.07 (− 0.08, 0.22)	0.3880
Q3	0.41 (0.28, 0.55)	< 0.0001	0.30 (0.16, 0.44)	< 0.0001	0.18 (0.01, 0.35)	0.0328
Q4	1.15 (1.02, 1.29)	< 0.0001	0.96 (0.83, 1.09)	< 0.0001	0.71 (0.47, 0.95)	< 0.0001
*P* for trend	< 0.001		< 0.001		< 0.001	

Model I adjust for age and gender

Model II model adjust for age, gender, SBP, DBP, HR, smoking, physical activity, hypertension, hyperlipidemia, stroke, CKD, race, TC, LDL-c, ALT, ALP

*Abbreviation*: *CI* Confidence interval

### Association between VAI and FPG in subgroups

After the correction of multiple potential confounding factors, the association between VAI and FPG levels remained significant in all subgroups stratified by age, gender, hypertension and hyperlipidemia. No significant association between VAI and FPG levels was found in diabetes subgroup in the fully adjusted model (β = 0.59, 95% CI: − 0.41-1.19, *p* = 0.0535). To further assess the impact of glucose-lowering drugs on FPG, the association between VAI and FPG was explored in diabetic patients receiving and not receiving antidiabetic treatment, respectively. The results showed that VAI was not associated with FPG in participants with diabetes no matter they received antidiabetic drugs or not (Table 4).

**Table 4:** Association between VAI and fasting plasma glucose, stratified by age, gender, diabetes, hypertension and hyperlipidemia

Exposure	N	β (95%CI)	*P* value	*P* for interaction
Stratified by age		0.47 (0.34, 0.60)		0.0019
< 60	3094	0.58 (0.36, 0.80)	< 0.0001	
≥ 60	1343		< 0.0001	
Stratified by gender				0.1066
Male	2175	0.63 (0.45, 0.82)	< 0.0001	
Female	2262	0.55 (0.41–0.63)	< 0.0001	
Stratified by diabetes				< 0.0001
Non-diabetes	3835	0.34 (0.27, 0.41)	< 0.0001	
Diabetes	602	0.59 (0.41–1.19)	0.0535	
Stratified by antidiabetic treatment				0.0826
Receiving antidiabetic treatment	500	0.61 (−0.31,1.52)	0.1953	
Not receiving antidiabetic treatment	102	−0.17 (−2.64, 2.31)	0.8948	
Stratified by hypertension				< 0.0001
Non-hypertension	2983	0.29 (0.18, 0.40)	< 0.0001	
Hypertension	1454	0.93 (0.68, 1.17)	< 0.0001	
Stratified by lipidemia				0.0012
Non-lipidemia	3058	0.31 (0.18, 0.44)	< 0.0001	
Lipidemia	1379	0.80 (0.60, 1.01)	< 0.0001	

Model adjust for age, gender, SBP, DBP, HR, smoking, physical activity, hypertension, hyperlipidemia, stroke, CKD, race, TC, LDL-c, ALT and ALP, except for the subgroup variable

### Nonlinear relationship exploration between VAI and FPG

**Fig. 2:**
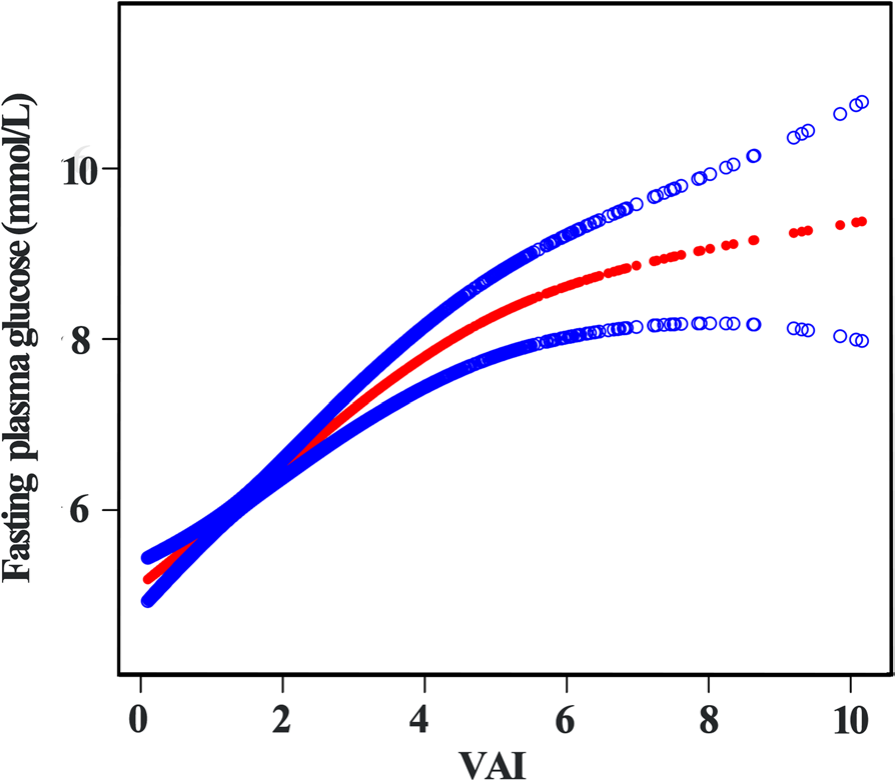
Association between VAI and fasting plasma glucose

**Fig. 3:**
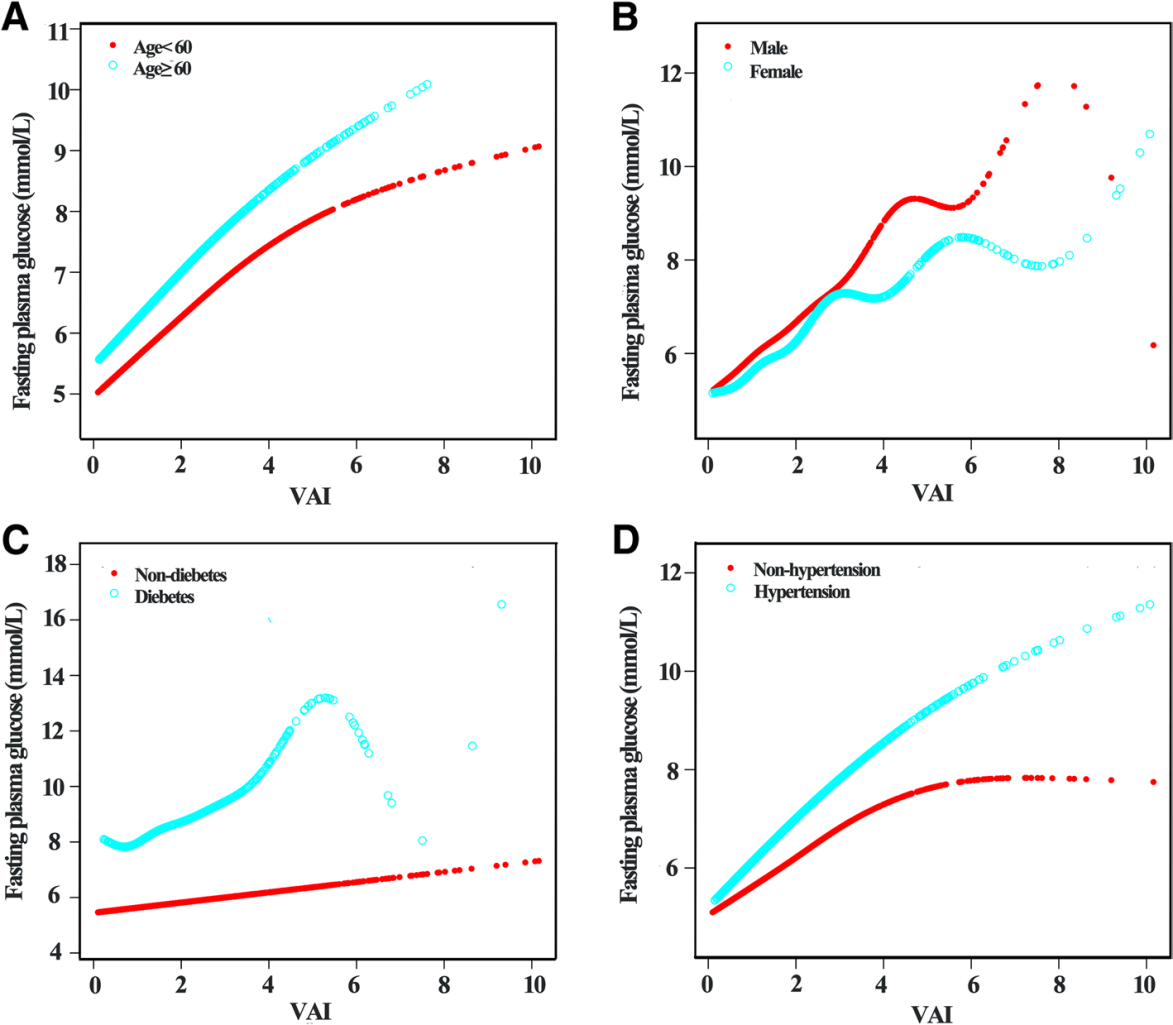
Association between VAI and fasting plasma glucose, stratified by age (**A**), gender (**B**), diabetes (**C**) and hypertension (**D**)

Generalized additive model (GAM) and smooth curve fitting analysis were performed to explore the nonlinear relationship between VAI and FPG. As presented in Fig. 2, a nonlinear association between VAI and FPG levels was observed after adjusting for age, gender, BMI, WC, SBP, DBP, HR, smoking, physical activity, hypertension, hyperlipidemia, stroke, CKD, race, TG, TC, HDL-c, LDL-c, ALT and ALP. FPG displayed an increasing trend with the increasement of VAI. Furthermore, threshold effect analysis was conducted using two-piecewise linear regression, and 4.02 was identified as the inflection point. We observed a dramatically positive correlation between VAI and FPG when VAI was below 4.02 (β = 0.73, 95% CI: 0.59–0.87, *p* < 0.0001), and the positive association between VAI and FPG also existed when VAI was higher than 4.02 (β = 0.23, 95% CI: 0.07–0.40, *p* = 0.0063) (Table 5). The smooth curve fittings in different subgroups stratified by age, gender, diabetes and hypertension were shown in Fig. 3. Similarly, non-linear relationship between VAI and FPG in subgroups were observed except for non-diabetic patients.

**Table 5:** Threshold effect analysis of VAI on fasting plasma glucose using piece-wise linear regression

Inflection point of VAI	β	*P* value
< 4.02	0.73 (0.59, 0.87)	< 0.0001
≥4.02	0.23 (0.07, 0.40)	0.0063
Log-likelihood ratio	< 0.001

### Receiver operating characteristic (ROC) analysis of VAI for predicting FPG elevation

**Fig. 4:**
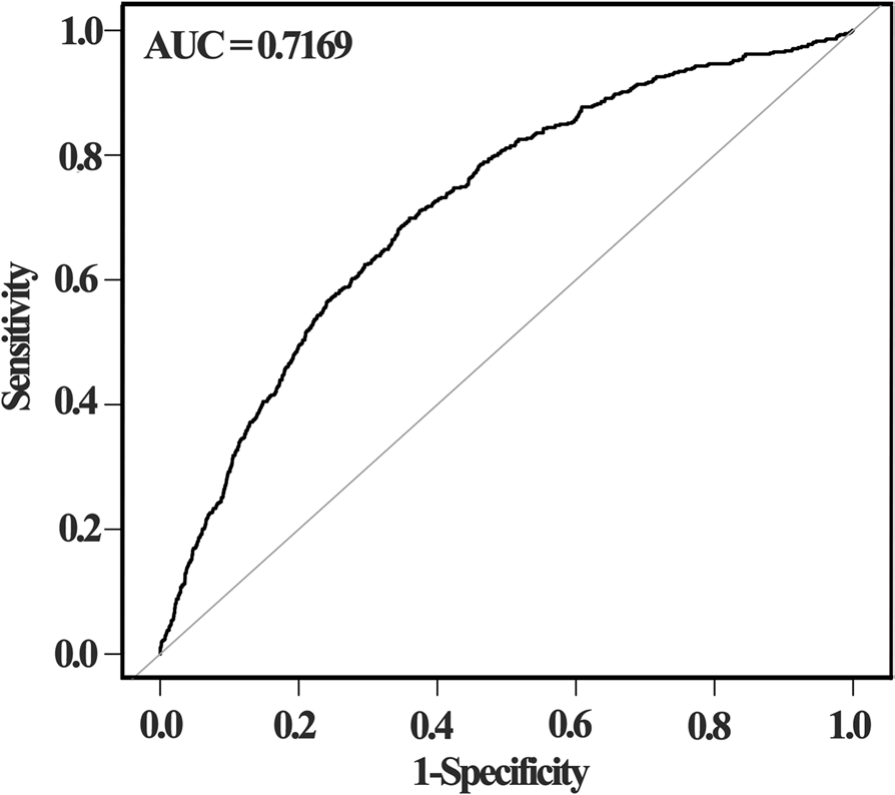
The ROC analysis of VAI for predicting FPG ≥ 7.0 mmol/L

To further explore the predictive value of VAI for FPG elevation, participants were divided into elevated FPG group (FPG ≥ 7.0 mmol/L) and control FPG group (FPG < 7.0 mmol/L) according to the definition of diabetes. ROC curve analysis was performed to explore the predictive value of VAI for identifying FPG ≥ 7.0 mmol/L (Fig. 4) after adjusting for age, gender, SBP, DBP, HR, smoking, physical activity, hypertension, hyperlipidemia, stroke, CKD, race, TC, LDL-c, ALT and ALP. The area under the curve (AUC) was 0.7169 (95% CI: 0.6948–0.7389), the cut-off value of VAI was 1.4315, and the corresponding sensitivity and specificity were 64.03 and 69.90%, respectively. Additionally, accuracy, positive and negative likelihood ratio were presented in Table 6.

**Table 6:** ROC performance of VAI for FPG ≥7.0 mmol/L

Variable	AUC	95%CI	Best threshold	Specificity	Sensitivity	Accuracy	Positive-LR	Negative-LR
VAI	0.7169	0.6948–0.7389	1.4315	0.6403	0.6990	0.648	1.9433	0.4701

## Discussion

The current study assessed the association between a novel indicator of adiposity distribution and dysfunction, VAI, and FPG. We found that VAI was positivity associated with FPG level based on a large-scale NHANES enrolling 4437 participants. Multiple linear regression analysis showed VAI was independently risk indicator for FPG. An increase of one unit in VAI was associated with 0.52 mmol/L higher FPG level after adjusting confounding factors. As a categorical variable, 4th VAI quartile group was associated with 0.71 mmol/L higher FPG level compared with 1st VAI quartile group. We also observed a non-linear association between VAI and FPG in the whole population. Additionally, VAI had a good predictive value for assessment of FPG elevation. Therefore, VAI acts as a simple and accessible parameter, participants with increased VAI level should be paid close attention to, and further FPG is recommended to determine the presence of dysglycemia.

Obesity is one of leading established risk factors for T2DM [32]. Specifically, individuals with adiposity distributed around visceral organs (abdominal adiposity) were regarded at higher risk for suffering insulin resistance and diabetes compared with those with subcutaneous adiposity [33, 34]. A study based on Mendelian randomization approach further provided a causal association between visceral adiposity and T2DM using a surrogate indicator termed WHRadjBMI [35]. It is well recognized that computer tomography and magnetic resonance imaging are gold standards for quantitative detection of visceral adipose tissue, however, they are not proper methods for research and clinical use due its inconvenience and expense [36]. Therefore, Amato et al. established a gender-specific indicator for visceral adiposity assessment termed VAI on the basis of WC, BMI, TG and HDL [19]. Subsequently, several researches have revealed the association between VAI and diabetes. Data from China Health and Nutrition Survey suggested VAI was positively associated with the risk for developing diabetes, and VAI had the highest predictive diagnostic ability for diabetes compared with BMI and WC [37]. A cohort study from Jiangsu revealed that patients with highest VAI had 2.55-fold risk of suffering diabetes [38]. However, it remains largely unknown on the relationship between VAI and FPG.

This is the first report showing VAI was non-linearly independently associated with FPG level, and 4.02 was identified as the inflection point. FPG dramatically went up with the increase of FPG, and FPG remained a relatively mild upward trend when VAI was higher than 4.02. Consistently, Zhou et al. investigated the prospective relationship between VAI and new-onset IFG in hypertensive patients, and reported a higher risk of new-onset IFG in patients with quartile 4 VAI compared with those in quartile 1–3 [39].

Another research conducted in Mexican population proved VAI was independently associated with IFG and VAI had highest AUC for predicting IFG compared with TG and waist-to-hip ratio [40]. In contrast, VAI was found not positively associated with FPG in previous research conducted among Chinese individuals. Furthermore, another two surrogate indices lipid accumulation product index and cardiometabolic index showed superior ability in predicting IFG to VAI [41]. These differences might be attributed to different research population. Li enrolled nonobese individuals without diabetes, whereas we included participants regardless of their diabetes status and their WC and BMI levels. Our subgroup analysis showed VAI was not an independent risk indicator for FPG in patients without diabetes, which was consistent with previous findings [41].

We observed that the positive relationship between VAI and FPG remained stable in participants regardless of age, gender, hypertension and hyperlipidemia, indicating VAI could reflect more detrimental mechanisms beyond these classic risk factors. The potential mechanisms underlying VAI and FPG might include the following aspects: Excessive visceral fat promoted the secretion of increased inflammatory adipokine, including IL-6 and leptin, which might contribute to the occurrence of insulin resistance and diabetes [42]. Nasser et al. reported VAI was the only determinant factor of adiponectin, which was regarded as the sole protective adipokine with anti-diabetogenic property [43]. Visceral lipid accumulation in adipocytes induced cellular stress and activation of JNK signaling, contributing to increased pro-inflammatory cytokines production and increased acute phase protein synthesis in adipose tissue, subsequently resulting in decrease of glucose uptake, esterification and storage of free fatty acid, eventually leading to dysglycemia [44].

Previous researches have explored the optimal cut-off value of VAI for metabolic syndrome (MetS) and diabetes. Chen suggested VAI was significantly associated with MetS, and a VAI of 2.282 was calculated to determine the occurrence of MetS in subjects with obstructive sleep apnea [45]. Similar results were found in Sara’s research, and the optimal cut-off value of VAI for MetS identification was 1.775 in whole obese population [46]. The optimal cut-off value of VAI was 1.52 for detecting prediabetes and diabetes (AUC = 0.687) in a German population [47]. To date, few publications have explored the predictive value of VAI for elevated FPG. According to our results of ROC analysis, VAI had a good predictive value for FPG elevation (AUC = 0.7169, 95% CI: 0.6948–0.7389), and the cut-off value of VAI was 1.4315. The predictive potential of VAI for FPG elevation still need further verifications from studies in different population.

A large sample size is a strength in our study. However, some limitations need to be addressed: Firstly, we can only provide the association but not a causal relationship between VAI and FPG because NHANES is a cross-sectional observational study. Secondly, the indispensable data for the calculation of VAI and FPG were absent for some individuals, therefore, a large number of participants were excluded in the research, which might lead to selection bias. Finally, the cohort might not be representative of general population because all included participants were American.

## Conclusions

In conclusion, the current study indicated a positively nonlinear relationship between VAI and FPG. VAI was an independent risk indicator for FPG and VAI had a good predictive value for elevated FPG. VAI might become a useful surrogate indicator for risk assessment and treatment of hyperglycemia in clinical practice.

## Data Availability

Publicly available datasets were analyzed in this study. This data can be found at: https://www.cdc.gov/nchs/nhanes/index.htm.
